# Are Meteorin-Like Peptide and Asprosin Important in the Diagnosis of Breast Tumors?

**DOI:** 10.7759/cureus.62979

**Published:** 2024-06-23

**Authors:** Nevin Kocaman, Elif Onat, Hilal Balta, Özlem Üçer

**Affiliations:** 1 Department of Histology and Embryology, Firat University Faculty of Medicine, Elazığ, TUR; 2 Department of Medical Pharmacology, Adıyaman University Faculty of Medicine, Adıyaman, TUR; 3 Department of Pathology, Fırat University Faculty of Medicine, Elazığ, TUR

**Keywords:** adipokine, asprosin, meteorin-like peptide, invasive ductal carcinoma, breast

## Abstract

Introduction: Breast cancer (BC) is one of the most common and leading causes of death in women. Therefore, early and accurate diagnosis is vital. In this study, meteorin-like (METRNL) peptide and asprosin levels were examined in breast tissue in invasive ductal carcinoma (IDC) of the breast, and the roles of these molecules in the diagnosis of BC were investigated.

Methods: In this retrospective study, tissues from patients with BC in the Pathology Department Laboratory of Fırat University Faculty of Medicine, Elazığ, Turkey, were used. Samples from 30 patients were used. The control group consisted of healthy breast tissues from the same patients. The pathology group consisted of breast tissues with IDC from the same patients. Breast tissue samples from both groups were evaluated immunohistochemically for METRNL and asprosin.

Results: Statistically significant differences were observed between both groups in terms of METRNL and asprosin. It was observed that METRNL and asprosin immunoreactivities were higher in breast tissues with IDC than in healthy breast tissues (p<0.001).

Conclusion: When the study results were evaluated, it was seen that there was a significant relationship between healthy breast tissues and the ones with IDC in terms of METRNL and asprosin. It is thought that both METRNL and asprosin may be really important in the future for the early diagnosis and treatment of BC.

## Introduction

Invasive ductal carcinoma (IDC) is the most common type of malignant breast cancer (BC), accounting for approximately 70-80% of invasive BCs. IDC has a heterogeneous structure in terms of clinical and histopathological features, prognosis, treatment, gene expression, and progression. Important proteomic determinants of progression from intraductal pre-invasive malignant lesions of the breast, such as ductal carcinoma in situ (DCIS), to IDC remain poorly defined and unvalidated [1]. The WHO has identified two different and related strategies to ensure an early diagnosis of cancer. They are the recognition of symptomatic cancer at an early stage and the identification of asymptomatic disease in apparently healthy individuals [2,3]. Therefore, studies on early diagnosis are a prerequisite for community-based screening, which will improve treatments for BC patients and ensure positive results.

Meteorin-like, also called METRNL, meteorin-β, subfatin, and cometin, is a recently discovered protein with pleiotropic properties in terms of inflammation, immunology, and metabolism. Previous research on this protein has examined its effects on energy consumption and glucose homeostasis. Various studies have attempted to define the molecular role of METRNL in glucose metabolism and obesity-related problems, but the results are clinically contradictory. In recent studies, METRNL's multifaceted protective properties in cardiometabolic diseases and the regulation of immune response such as stimulating macrophage activation, angiogenesis, tissue restructuring, bone formation, and preventing dyslipidemias have begun to be discovered. Understanding the properties of this protein is important in explaining its therapeutic aspect and its importance in the diagnosis of some diseases [4].

Asprosin, a glucogenic adipokine associated with fasting, was first identified by Romere in 2016 [5]. By interacting with the G protein-coupled receptor called OR4M1, asprosin promotes hepatic glucose release in the liver and appetite stimulation in the hypothalamus through cyclic adenosine monophosphate (cAMP) signaling activation. Many studies show that asprosin plays a role in the formation and progression of various diseases such as diabetes, obesity, cardiomyopathy, cancer, and polycystic ovary syndrome. It is involved in the regulation of various cellular and physiological processes such as appetite stimulation, glucose and insulin release, apoptosis, and inflammatory response [6].

In this study, we tried to investigate whether these proteins have a role in the diagnosis of IDC by examining METRNL and asprosin levels in breast IDC tissue.

## Materials and methods

Research and publication ethics 

The study was approved by the Fırat University Local Ethics Committee (approval Number: 2024/03-49). The samples were taken from the Department of Pathology, Fırat University Faculty of Medicine, Elazığ, Turkey. Patients, none of whom had known familial cancer predisposition syndromes or other unrelated cancer diagnoses, were included in our study. Biopsy samples were taken from patients who had not received radiotherapy or chemotherapy or any topical treatment. Two groups were created, each consisting of 30 samples. The control group consisted of samples taken from healthy breast tissues of the same patients. The other group consisted of samples taken from the same patients' tissues with IDC. Fine needle aspiration samples and all slides of mastectomy material from patients diagnosed with IDC were reviewed by two pathologists and one histologist, all of whom were blinded. The results were evaluated by comparing METRNL and asprosin levels in the tissue samples of the groups.

Immunohistochemistry

We cut 4-5 µm sections from the paraffin-embedded specimens and mounted them on poly-L-lysine-coated glass slides. Deparaffinized specimens were rehydrated through a graded alcohol series and heated in a microwave oven at 750 W for 12 (seven + five) minutes in citrate buffer, pH 6.0, for antigen retrieval [7]. After heating, the specimens were cooled at room temperature for 20 minutes, rinsed with phosphate-buffered saline (PBS) (P4417; Sigma-Aldrich, St. Louis, Missouri, United States) three times for five minutes each, then incubated with hydrogen peroxide for five minutes to block endogenous peroxidase activity (Hydrogen Peroxide Block, TA-125-HP; Lab Vision Corp., New York City, New York, United States). Ultra V Block (TA-125-UB; Lab Vision Corp.) was added for five minutes to prevent background staining, and then the specimens were incubated with an ASP (anti-asprosin antibody, FNab09797; Fine Biotech Co., Wuhan, China) and METRNL antibody (anti-METRNL antibody, MBS7004241; MyBioSource, San Diego, California, United States) diluted 1:200 for 60 minutes in a humid chamber at room temperature.

Following incubation with primary antibodies, the specimens were rinsed with PBS three times for five minutes each, then incubated with biotinylated goat anti-polyvalent secondary antibody (anti-mouse/rabbit IgG) (TP-125-BN; Lab Vision Corp.) in a humid chamber at room temperature for 30 minutes. The sections were washed three times with PBS and then incubated with streptavidin peroxidase (TS-125-HR; Lab Vision Corp.) for 30 minutes. Drops of 3-amino-9-ethylcarbazole (AEC) substrate + AEC chromogen solution (AEC substrate, TA-015-HAS; AEC Chromogen, TA-002-HAC; Lab Vision Corp.) were applied and the tissues were washed with PBS after labeling was checked by light microscopy. Mayer’s hematoxylin was used for counterstaining. Sections were rinsed in PBS and distilled water and then mounted with a mounting medium (Large Volume Vision Mount, TA-125- UG; Lab Vision Corp.). Mounted sections were examined and photographed using a Leica DM500 microscope (Leica DFC295; Berlin, Germany). We scored the distribution of staining as 0.1, < 25%; 0.4, 26-50%; 0.6, 51-75%; and 0.9, 76-100%, and the intensity of staining as 0, no staining; 0.5, very little staining; 1, little staining; 2, moderate staining; and 3, very strong staining. A histoscore was calculated as distribution × intensity [8].

Statistical analysis

The statistical analyses were conducted using IBM SPSS Statistics for Windows, Version 22, (Released 2013; IBM Corp., Armonk, New York, United States). The One-Way ANOVA test was employed, and post-hoc multiple comparisons were carried out using Tukey's HSD (Honestly Significant Difference) test. Kolmogorov Smirnov test was used for the normal distribution test. Results are presented as mean ± SD, with a significance level set at p<0.05, indicating statistical significance.

## Results

Immunohistochemical findings

**Figure 1:**
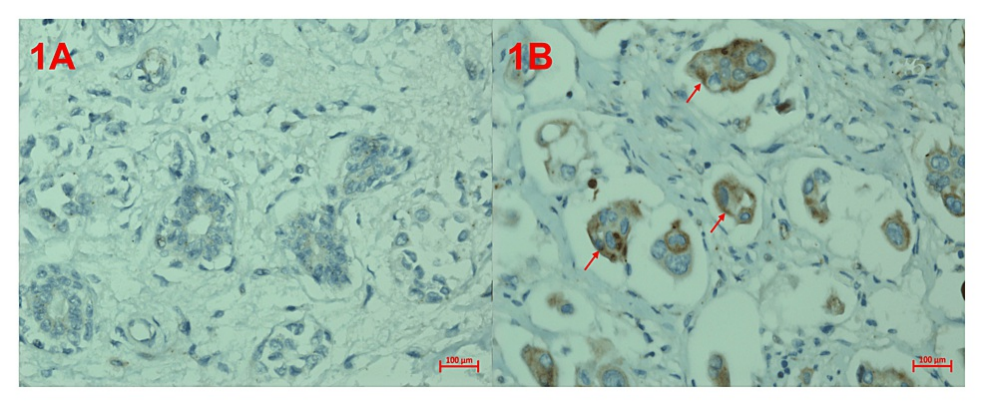
Immunohistochemical analysis of METRNL protein in the breast (A) Control METRNL immunoreactivity, (B) breast cancer METRNL immunoreactivity. METRNL: meteorin-like

**Figure 2:**
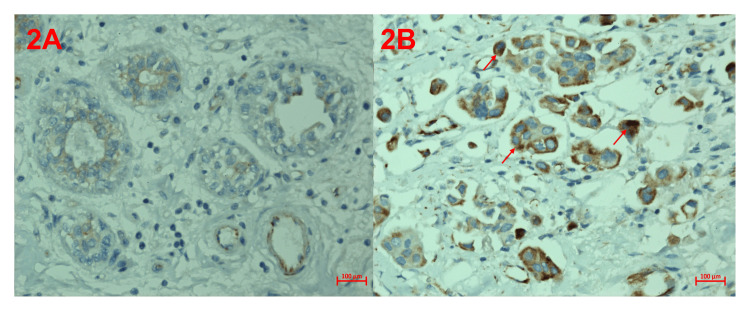
Immunohistochemical analysis of asprosin protein in the breast (A) Control asprosin immunoreactivity, (B) breast cancer asprosin immunoreactivity.

Breast tissue samples from patients with IDC were stained immunohistochemically with METRNL and asprosin. Histoscore was created based on scoring based on the extent and intensity of staining. The groups were compared among themselves in terms of METRNL and asprosin expression. The preparations were evaluated and photographed under a Zeiss Axio Scope A1 microscope (Carl Zeiss AG, Oberkochen, Germany) (p < 0.001) (Table 1) (Figures 1-2 ).

**Table 1:** METRNL and asprosin immunoreactivity histoscore Values are given as mean ± standard deviation. ^a ^Compared with control (p<0.001). METRNL: meteorin-like

Group	Control	Breast cancer
METRNL	0.008 ± 0.019	0.96 ± 0.23^a^
Asprosin	0.25 ± 0.12	2.1 ± 0.53^a^

METRNL and asprosin immunoreactivity

METRNL and asprosin proteins were stained cytoplasmically in breast tissue samples from patients with IDC. In IDC tissues, METRNL and asprosin proteins were found to be immunoreactive in both the cytoplasm and membranes of the cells.

While the expression of METRNL protein was highest in IDC breast tissues, it was detected less in healthy breast tissues (Table 1). It was determined that the expression difference between the groups was statistically significant (p<0.001). The histoscores depicting METRNL immunoreactivity for the two groups are illustrated in Figure 1.

As seen in Table 1, asprosin expression was detected in breast tissues with IDC. Asprosin expression was observed to be higher in IDC breast tissues and less in healthy breast tissues (Table 1). When asprosin expression was compared between groups, it was observed that it differentiated IDC breast tissue from healthy breast tissue and the difference between them was statistically significant (p<0.001). The histoscores representing asprosin immunoreactivity for the two groups are depicted in Figure 2.

## Discussion

There are rapid advances in the diagnosis and treatment of breast IDC. Despite this, current DCIS treatment options are still suboptimal due to the lack of full understanding of the underlying mechanisms of the existing pathology. Imaging methods used in the differential diagnosis of DCIS and early-stage IDC are inadequate. However, it is difficult to predict whether DCIS will turn into IDC. The mechanisms that cause progression from DCIS to IDC are still not fully understood [9]. Based on this problem, in this study, the roles of these proteins in the diagnosis of breast IDC were investigated by examining the presence and level of METRNL and asprosin in the neoplastic tissues of the breast with IDC. As a result of the study, a significant relationship was discovered between healthy breast tissue and IDC in terms of METRNL and asprosin levels, and it was concluded that these proteins may be surrogates of IDC.

In patients with endometrial adenocarcinoma, METRNL expression was observed to be increased in carcinoma tissue compared to healthy tissue [10]. In another study, it was found that METRNL expression increased as atypia increased in tissue with endometrial hyperplasia, and the highest level was in endometrial adenocarcinoma (EAC) [11]. Studies investigating the relationship between METRNL and cancer show that METRNL has a protumor effect in pancreatic cancer and emphasize that it is diagnostically valuable for bladder cancer, basal cell cancer of the skin and malignant mesothelioma [12,13-15]. Recent developments indicate that METRNL plays an oncogenic role in cellular regulation in colorectal cancer [16] and is present in colorectal adenocarcinoma [17]. It is also thought that overproduction of METRNL may be associated with advanced colorectal cancer and poor prognosis. This may be clinically important for the progression of colorectal cancer [18]. In this study conducted in breast tissue with IDC, the increase in METRNL compared to healthy tissue supports the idea of METRNL being an important biomarker in the diagnosis of cancer. However, in order to make a definitive comment on this issue, more clinical studies are needed to elucidate the mechanism of action of METRNL.

Studies, although few in number, show that asprosin is produced in various types of human cancer [6]. This conclusion was once again reached in this study, where asprosin levels were observed to be increased in breast IDC tissues. In another study conducted in patients with colon carcinoma, it was observed that asprosin expression was increased in carcinoma tissues compared to healthy tissues [19]. Asprosin is thought to be a promising marker for the early diagnosis of pancreatic cancer [20]. Kocaman and Artaş [21] found that immunoreactivity was more severe in prevalence and intensity than reactive mesothelial hyperplasia. In ovarian cancer, it has been found that asprosin is expressed at different rates in different histological types [22]. Apoptosis is a natural defense system against neoplastic growth [23]. Many studies show that asprosin plays a role in regulating the apoptosis mechanism at the cellular level. It has been observed that asprosin, in particular, is effective in the apoptosis of mesenchymal stromal cells by regulating the ERK1/2-SOD2 pathway [24]. Additionally, high asprosin levels reduced the apoptotic death of cardiac microvascular endothelial cells [5]. Contrary to these studies, asprosin expression was observed to decrease in patients with endometrial carcinoma [10]. It is thought that asprosin has an important effect on carcinogenesis, but there are few studies on the role of asprosin in cancer and apoptosis, making it difficult to comment on this subject. Therefore, more detailed studies are needed [6].

Limitations

One of the most important limitations of this study is that although tissue samples with IDC have been studied, tissue samples with DCIS have not been studied. Although it is known that METRNL and asprosin are increased in some cancers, more studies are needed to elucidate their mechanism of action because they are new molecules. In addition, other clinical characteristics of the patients may need to be taken into account in order for METRNL and asprosin to be accepted as biomarkers in cancer diagnosis.

## Conclusions

In conclusion, the significant changes in METRNL and asprosin levels in IDC of the breast compared to healthy breast tissues suggest that these molecules may play a role in the pathophysiology of IDC. With a clearer understanding of the molecular mechanisms of METRNL and asprosin, they may be promising in the diagnosis and treatment of IDC in the future. However, for this, many more preclinical studies need to be conducted and the results obtained must be supported by clinical data.
